# Renal function trajectories in hepatitis C infection: differences between renal healthy and chronic kidney disease individuals

**DOI:** 10.1038/s41598-021-96782-x

**Published:** 2021-08-25

**Authors:** Cheng-Kai Hsu, Tai-Shuan Lai, Yih-Ting Chen, Yi-Ju Tseng, Chin-Chan Lee, Chun-Yu Chen, Heng-Jung Hsu, Heng-Chih Pan, Li-Wei Chen, Cheng-Hung Chien, Chih-Lang Lin, Rong-Nan Chien, I-Wen Wu

**Affiliations:** 1 Department of Nephrology, Chang Gung Memorial Hospital, 222, Mai-Chin Road, Keelung, 20401 Taiwan; 2 Division of Nephrology, Department of Internal Medicine, National Taiwan University Hospital, National Taiwan University, Taipei, Taiwan; 3 Department of Information Management, National Central University, Taoyüan, Taiwan; 4 Department of Gastroenterology and Hepatology, Keelung Chang Gung Memorial Hospital, Keelung, Taiwan; 5 Community Medicine Research Center, Chang Gung Memorial Hospital, Keelung, Taiwan; 6 College of Medicine, Chang Gung University, Taoyüan, Taiwan

**Keywords:** Chronic kidney disease, Liver diseases

## Abstract

Associations between hepatitis C virus (HCV) and chronic kidney disease (CKD) have been reported; however, differences of renal progression between general and CKD population remain to be elucidated in prospective studies. A total of 1179 participants, who have tested for anti-HCV antibody, were enrolled and prospectively followed for 3 years. The risks associated with HCV infection, in terms of incidence of CKD, annual estimated glomerular filtration rate (eGFR) changes and 50% decline of eGFR at 3-year from baseline, were compared between normal renal function subjects and CKD patients. Overall, 111 of 233 (47.6%) CKD patients and 167 of 946 (17.7%) non-CKD subjects had HCV infection. The crude incidence rates of CKD were 226.9 per 1000 person-years and 14.8 per 1000 person-years in in HCV and non-HCV infected patients, respectively. The adjusted hazard ratio of HCV infection for incident CKD was 7.9 (95% CI 5–12.7). The HCV-infected normal renal function subjects were independently associated with increased risks of eGFR decline in the 1-year, 2-year and 3-year, respectively. The risk associations remained significant in 50% decline of eGFR at 3 years models and in different subgroup analyses. The increases of risks of eGFR decline were also notorious among overall HCV-infected CKD patients. However, the risk associations were less prominent in subgroup analyses (elderly, women and diabetic patients). The findings highlighted the importance of viral diagnosis with not only prognostic but also public health implications for preserving kidney function.

## Introduction

Understanding of risks of chronic kidney disease (CKD) is important, since the disease is associated with comorbidities, mortality and financial burden. Conventional risk factors for CKD include age, male gender, proteinuria, cardiovascular disease, smoking, diabetes and hypertension^1^. In addition, several non-traditional risk factors have been identified^2^. Emerging evidences have demonstrated possible associations between hepatitis C virus (HCV) and renal disease^3–10^. Chronic HCV infection can affect kidney function either through systemic inflammation or by increasing risk factors of CKD, including metabolic syndrome, diabetes mellitus, mixed cryoglobulinemia, membranoproliferative glomerulonephritis, cardiovascular disease, lymphoproliferative disorders and depression^11–14^.

The prevalence of HCV infection is approximately 1% in the western population affecting more than 71.1 million people globally^15,16^. However, the prevalence of this infection is higher than the burden of diabetes mellitus, in some endemic Asian countries, such as Taiwan, having a prevalence rate ranged from 1.6 to 19.6%^17^. This viral disease can increase morbidity, medical cost, impaired quality of life, high mortality and deserves timely intervention^11,18,19^.

A number of researches have indicated controversial associations between chronic HCV infection and development of CKD or renal progression. Various cohort studies and meta-analyses have shown that HCV-infected patients were associated with greater risks of developing CKD, progressive renal deterioration^3,20^ and end stage renal disease than patients without HCV infection^4–6,21–23^. However, other studies found that carriers of HCV infection were associated with proteinuria rather than reduced eGFR (estimated glomerular filtration rate)^24,25^. Furthermore, Rogal et al. concluded that HCV infection was not significantly associated with progressive CKD (defined as decline of eGFR of ≥ 30 mL/min/1.73 m^2^ from baseline)^26^. As such, the exact roles of HCV infection played on the deterioration of renal function remain to be elucidated.

Therefore, the aims of this prospective cohort study were to determine the incidence of CKD associated with HCV infection among normal renal function subjects and to compare the changes of eGFR trajectories associated with HCV infection among individuals having normal renal function or CKD.

## Materials and methods

### Study design and patient settings

This prospective cohort study enrolled participants of multidisciplinary education program of CKD Prevention Center of Department of Nephrology of Chang Gung Memorial Hospital at Keelung. This cohort included CKD patients of nephrology outpatient clinics as well as habitants of neighborhood community of Northwestern Taiwan, recruited from health promotion program. All participants aged ≥ 18 years having tested for anti-HCV antibody (Ab) during January 2012 to December 2014 were included for study. Those patients who underwent dialysis therapy, renal transplant graft or prior treatment for HCV infection were excluded from study. The serum creatinine was checked at annual-basis for consecutive three years to denote changes of renal function. Patients with incomplete laboratory data were excluded from analysis. Patients, who die or lose to follow-up, were censored. This study was conducted in adherence to the Declaration of Helsinki and approved by the Institutional Review Board at Chang Gung Memorial Hospital (IRB No. 201800273B0C602, 101-5105B, 201902192B0, clinical trial registration No. NCT04300387). The informed consent was obtained from all patients.

### Ascertainment of HCV hepatitis

The anti-HCV Ab was measured at baseline by using a third-generation enzyme immunoassay kit (AxSYM® HCV Version 3.0; Abbott Laboratories, Abbott Park, IL, US). A value of 0.9 cutoff index was defined as positive.

### Outcomes of interest and follow-up

The CKD was defined as having eGFR ⩽ 60 mL/min/1.73 m^2^ or any urine protein/creatinine ratio (UPCR) ≥ 150 mg/g in 2 separate occasions three months apart. The eGFR was calculated by Chronic Kidney Disease Epidemiology Collaboration equation (CKD-EPI)^27^. Annual changes in eGFR (mL/min/1.73m^2^) were recorded for every patient. The developments of new CKD (incident CKD model), absolute/relative changes of eGFR and percentages of patients having 50% decline of eGFR at 3rd years from the baseline (eGFR decline model) were accurately recorded during the follow-up period.

### Statistical analysis

Categorical variables were presented as frequency and percentage and compared using chi-square test or Fisher’s exact test. Continuous variables were expressed as means ± standard deviation (SD) or median (interquartile range, IQR) and compared using Student t-test or Mann Whitney U test. Kolmogorov-Simirnov method was used to test normality of numerical variables. Analysis of CKD event-free survival was derived from the Kaplan–Meier analysis. The Cox proportional hazards model was applied to estimate multivariable-adjusted hazard ratios (HRs) and 95% confidence intervals (CIs) of CKD associated with HCV infection in normal renal function population. Linear regression analyses were performed to examine the relationship between changes in eGFR and HCV status. Firth logistic regression model was conducted to estimate odd ratios (ORs) of 50% decline of eGFR from baseline value during follow-up^28^. Statistical analyses were performed with the Statistical Package for the Social Sciences version 21.0 (SPSS, Inc., US). All statistical tests were two-tailed and a *p*-value < 0.05 was considered statistically significant.

### Ethics approval and patient consent statement

This study was conducted in adherence to the Declaration of Helsinki and approved by the Institutional Review Board at Chang Gung Memorial Hospital (IRB No. 201800273B0C602, 101-5105B, 201902192B0, clinical trial registration No. NCT04300387. The informed consent was obtained from all patients.

## Results

### Baseline characteristics of HCV and non-HCV patients

**Figure 1:**
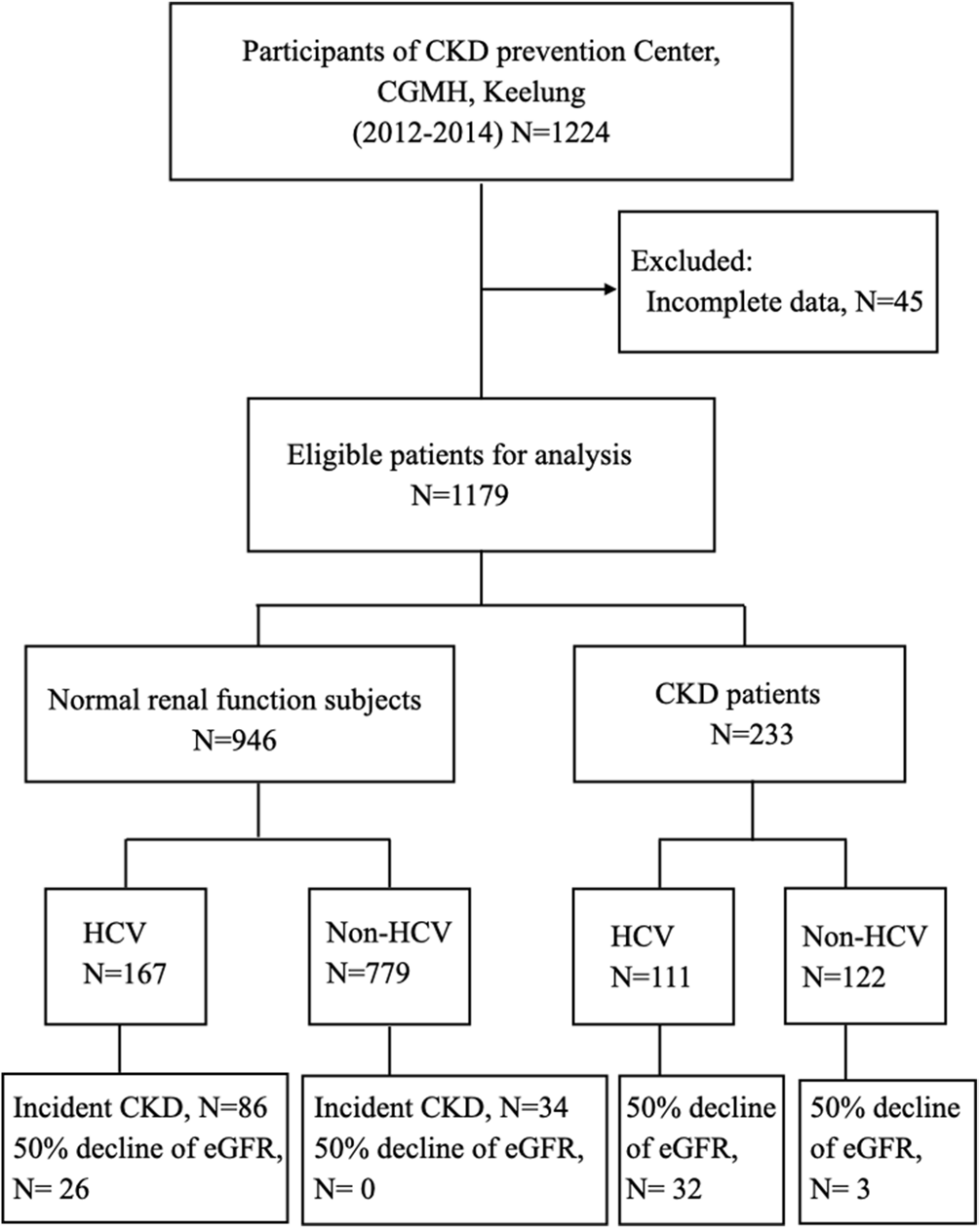
Study design and patient flow schema. CKD, chronic kidney disease; eGFR, estimated glomerular filtration rate; HCV, hepatitis C virus infection.

A total of 1179 participants (946 non-CKD and 233 CKD patients) were included for study from January 2012 and December 2014 (Fig. 1). The mean age of participants was 59.2 years and 400 (33.9%) were men. Table 1 summarizes the baseline characteristics between HCV (n = 278) and non-HCV infected subjects (n = 901), stratified by CKD status. The numbers of HCV infection were 111 (47.6%) among CKD patients and 167 (17.7%) among non-CKD subjects, respectively (*p* < 0.005). HCV-infected patients tended to be older, were more likely to be men, were more likely to have diabetes and hypertension than non-HCV subjects. HCV-infected patients also had lower eGFR, higher UPCR, lower serum level of albumin and hemoglobin but higher levels of aspartate aminotransferase, alanine aminotransferase, uric acid and glycated hemoglobin than those patients without HCV infection.

**Table 1:** Demographic characteristics of all patients stratified by CKD and HCV infection (n = 1179).

Variables	All (n = 1179)	Non-CKD (n = 946)			CKD (n = 233)		
		Non-HCV	HCV	*p*-value	Non-HCV	HCV	*p*-value
		(n = 779)	(n = 167)		(n = 122)	(n = 111)	
Age (years)	59.2 ± 11	56.7 ± 10.2	61 ± 10.7	< 0.001	64 ± 10.4	68.3 ± 10.6	0.002
Male, No (%)	400 (33.9)	240 (30.8)	69 (41.3)	0.009	39 (32)	52 (46.8)	0.02
Diabetes, No (%)	239 (20.3)	65 (8.3)	72 (43.1)	< 0.001	39 (32)	63 (56.8)	< 0.001
Hypertension, No (%)	374 (31.7)	180 (23.1)	51 (30.5)	0.043	69 (56.6)	74 (66.7)	0.114
eGFR (ml/min/1.73m^2^)	90 ± 27.7	98.2 ± 21.2	89.1 ± 22.4	< 0.001	81.4 ± 32.1	43.6 ± 20.6	< 0.001
AST (U/L)	27.3 ± 21.3	24.3 ± 15.5	47.8 ± 34.8	< 0.001	26.5 ± 9.1	50.2 ± 58.4	< 0.001
ALT (U/L)	29.2 ± 30.9	25.1 ± 22.8	49.2 ± 45.4	< 0.001	27.7 ± 16.4	50.4 ± 71.6	0.001
Uric acid (mg/dL)	5.6 ± 1.4	5.4 ± 1.3	6.1 ± 1.5	< 0.001	6.1 ± 1.5	6.8 ± 1.7	0.009
Albumin (g/dL)	4.6 ± 0.4	4.7 ± 0.3	4.3 ± 0.7	< 0.001	4.6 ± 0.3	3.8 ± 0.6	< 0.001
Hemoglobin (g/dL)	13.6 ± 1.6	13.8 ± 1.4	12.9 ± 2.1	< 0.001	13.6 ± 1.5	11.9 ± 1.9	< 0.001
Cholesterol (mg/dL)	209.1 ± 37.9	211.1 ± 36.3	202.4 ± 43.2	0.06	205.8 ± 41.9	193.1 ± 39.1	0.08
LDL (mg/dL)	126.2 ± 31.8	127.3 ± 31.1	125.8 ± 40.1	0.771	120.4 ± 32.6	107.2 ± 28.5	0.266
Triglycerides (mg/dL)	123.2 ± 88.6	118 ± 80.7	120.3 ± 57.6	0.827	157.4 ± 135.1	124.8 ± 79.6	0.134
Glycohemoglobin (%)	6 ± 1	5.8 ± 0.6	7.1 ± 1.8	< 0.001	6.4 ± 1.3	7.1 ± 1.8	0.01
UPCR, median (IQR) (mg/g)	64.1 (48–98.9)	56.6 (44–76.9)	90.8 (58.7–125)	< 0.001*	110.9 (73.1–279.4)	400.3 (111.4–924.7)	< 0.001*

*ALT* alanine aminotransferase, *AST* aspartate aminotransferase, *CKD* chronic kidney disease, *eGFR* estimated glomerular filtration rate, *HCV* hepatitis C virus, *IQR* interquartile range, *LDL* low-density lipoprotein, *UPCR* urine protein to creatinine ratio.

*p-value using Mann–Whitney U test.

### HCV and incident CKD

**Figure 2:**
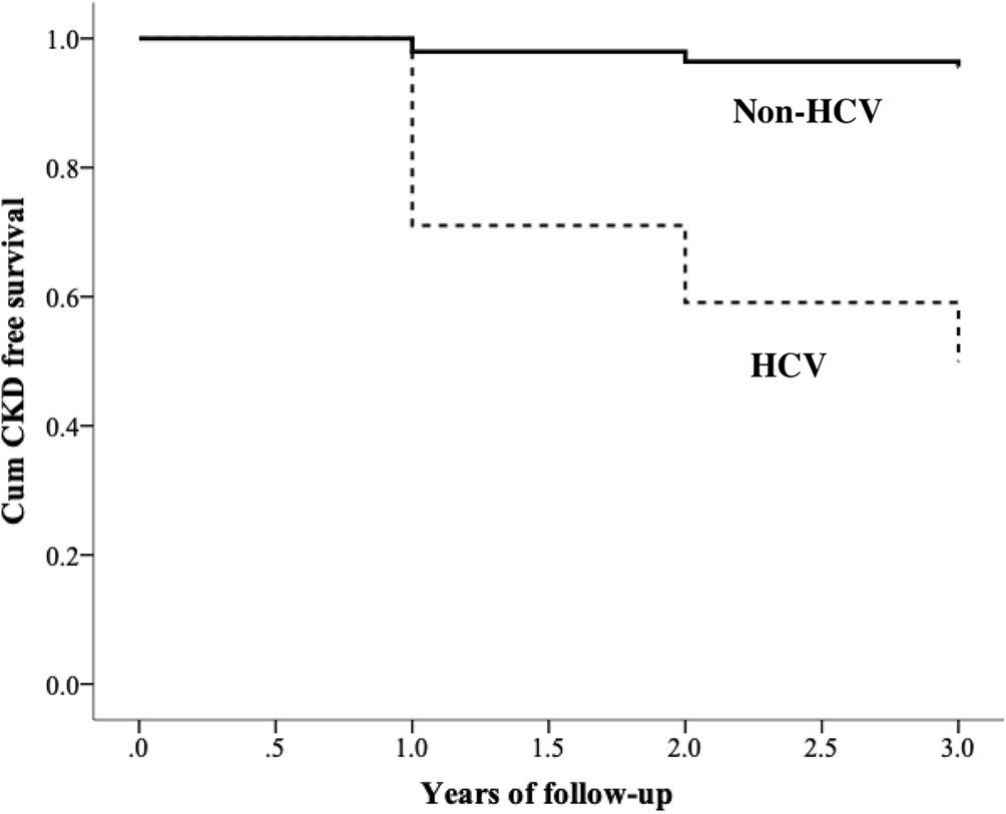
Cumulative proportion of CKD free survival by HCV infection at study entry. CKD, chronic kidney disease; HCV, hepatitis C virus infection. Log-rank test, *p* < 0.001.

From 949 non-CKD subjects, 86 of 167 HCV patients (51.5%) and 34 of 779 non-HCV patients (4.3%) developed incident CKD after mean follow-up of 36 months (*p* < 0.001). The crude incidence rates of CKD were 226.9 per 1000 person-years and 14.8 per 1000 person-years in HCV and non-HCV patients, respectively (Table 2). The Cox proportional hazards regression model revealed that presence of HCV infection had a 7.9-fold risk (HR 7.9; 95% CI 5–12.7) of developing CKD after adjusting for age, gender, hypertension, diabetes, baseline renal function and UPCR (Table 2). In subgroup analyses, we found that the adjusted HRs for development of CKD associated with HCV infection were higher for young adults (< 60 years), men, non-diabetes and non-hypertensive patients compared with elderly, women, diabetes and hypertensive patients (Table 2). Figure 2 illustrates that cumulative proportion of CKD event-free survival was lower in HCV group compared with non-HCV group (Log-rank test, *p* < 0.001).

**Table 2:** Incidence rates and hazard ratio (HR) for CKD according to HCV infection.

Population	HCV status	No. of patients	Person years	No. of CKD events	Time to CKD events	Crude incidence of CKD*	Crude HR (95% CI)	Adjusted HR^a^ (95% CI)
All patients	Non-HCV	779	2291	34	35.3	14.8	1	1
	HCV	167	379	86	27.2	226.9	16 (11–24)	7.9 (5–12.7)
**Age, years**								
18–59	Non-HCV	465	1383	7	35.7	5.1	1	1
	HCV	73	175	33	28.8	188.6	39 (17–88)	21.7 (8.8–53.8)
≥ 60	Non-HCV	314	908	27	34.7	29.7	1	1
	HCV	94	204	53	26	259.8	9.3 (5.8–15)	5.2 (2.9–9.2)
**Gender**								
Men	Non-HCV	240	704	13	35.2	18.5	1	1
	HCV	69	144	44	25	305.6	18 (9.7–34)	13.9 (6.7–29.1)
Women	Non-HCV	539	1587	21	35.3	13.2	1	1
	HCV	98	235	42	28.8	178.7	14 (8.3–24)	4.8 (2.6–9)
Diabetes	Non-HCV	65	178	10	32.9	56.2	1	1
	HCV	72	149	48	24.8	322.1	6.2 (3.2–12)	5.2 (2.1–12.9)
Non-diabetes	Non-HCV	714	2113	24	35.5	11.4	1	1
	HCV	95	230	38	29.1	165.2	15 (9–25)	8.8 (5–15.4)
HTN	Non-HCV	180	512	21	34.1	41	1	1
	HCV	51	108	26	25.4	240.7	5.2 (2.9–9.3)	5 (2.4–10.4)
Non-HTN	Non-HCV	598	1776	13	35.7	7.3	1	1
	HCV	117	274	60	28.1	218.9	29 (15.9–53)	12.5 (6.2–25.2)

Time to events was expressed in means (months). *Per 1000 person-years.

*CKD* chronic kidney disease, *HCV* hepatitis C virus, *HR* hazard ratio, *HTN* hypertension.

^a^Adjusted for age, gender, hypertension, diabetes mellitus, baseline eGFR, and urine protein to creatinine ratio.

### HCV and eGFR trajectory in normal renal function subjects

**Figure 3:**
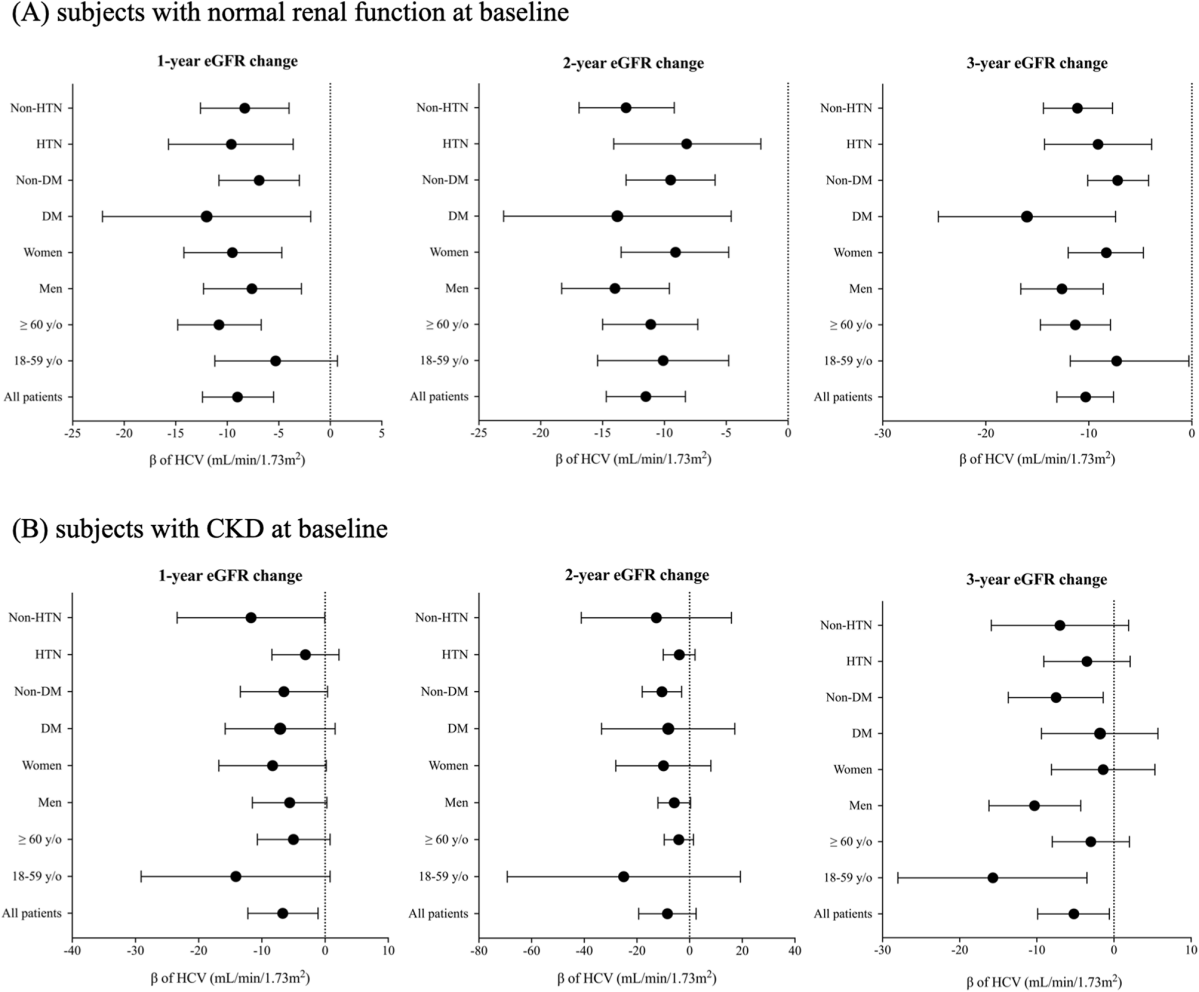
Unstandartized coefficients of HCV in a linear regression model for annual eGFR decline in subjects with normal function at baseline (**A**) and patients with CKD at baseline (**B**). CKD, chronic kidney disease; DM, diabetes mellitus; eGFR, estimated glomerular filtration rate; HCV, hepatitis C virus infection; HTN, hypertension.

Linear regression analysis was further conducted to explore the differences of eGFR trajectories in patients with HCV infection, by using change of eGFR at 12 month (first-year eGFR change: 12mo − 0mo), 24 month (second-year eGFR change: 24mo − 0mo) and 36 month (third-year eGFR change: 36mo − 0mo) versus the baseline eGFR. Figure 3 displays the β of HCV associated with eGFR changes, stratified by year and by comorbidities. Among all of normal renal function subjects, HCV-infected patients had more prominent eGFR declines than those without HCV infection (Fig. 3A, Table S1). The HCV infection was independently associated with increases of risks of eGFR decline, being the β of − 9 (95% CI − 12.4 to − 5.5, *p* < 0.001), − 11.5 (95% CI − 14.7 to − 8.3, *p* < 0.001), and − 10.3 (95% CI − 13.1 to − 7.6, *p* < 0.001), after adjusting for age, gender, hypertension, diabetes, baseline eGFR and UPCR, in the 1-year, 2-year, and 3-year linear regression models, respectively. The risk associations between eGFR decline and HCV infection remained significant in different subgroup analyses (Fig. 3A).

### HCV and eGFR trajectory in CKD patients

The increases of risks of eGFR declines also were notorious among overall CKD patients infected with HCV (Fig. 3B, Table S2). Linear regression analyses showed that HCV infection was independently associated with increased risks of eGFR decline after adjusting for age, gender, hypertension, diabetes, baseline eGFR and UPCR, except the 2-year model [β were − 6.7 (95% CI − 12.2 to − 1.1, *p* = 0.019), − 8.4 (95% CI − 19.3 to 2.5, *p* = 0.129) and − 5.2 (95% CI − 9.9 to − 0.6, *p* = 0.028) in 1-year, 2-year, and 3-year models, respectively, Fig. 3B). In contrast to the normal renal function subjects, the increases of risks of renal progression at 1st, 2nd or 3rd years were inconsistent in different subgroups of HCV infected-CKD patients stratified by age (< or ≥ 60 years old), gender, diabetic and hypertension status. The risks of annual eGFR decline associated with HCV were less prominent for women, diabetic patients and older patients during 3-year follow up (Fig. 3B). The differences of annual absolute (and relative) eGFR changes between HCV and non-HCV patients were illustrated in Table S1 (normal renal function subjects) and Table S2 (CKD patients), respectively.

**Figure 4:**
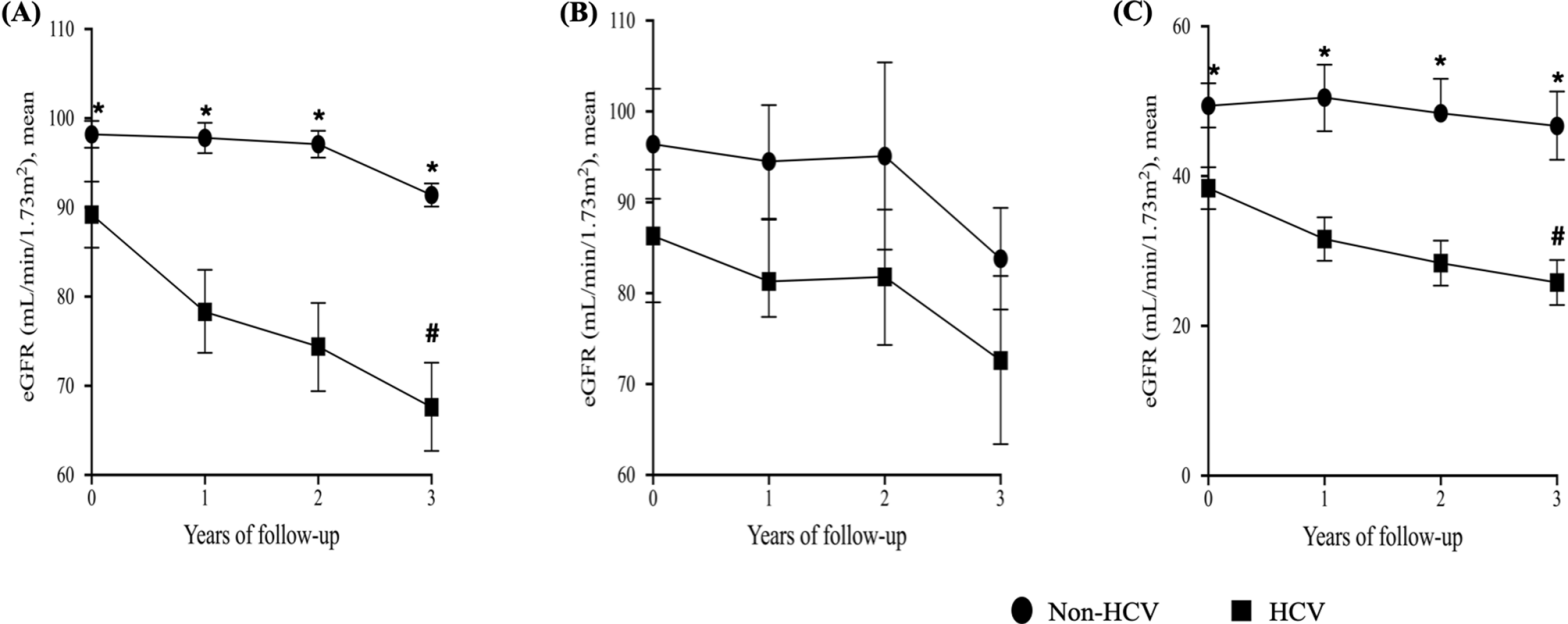
Differences of eGFR trajectories during follow-up according to HCV infection among normal renal function individuals and those with different stages of CKD. The mean slope of eGFR decline was − 6.56 mL/min/1.73 m^2^/year vs. − 2.27 mL/min/1.73 m^2^/year (*p* < 0.001) in HCV infected and non-HCV infected normal renal function subjects (**A**); − 4.85 mL/min/1.73 m^2^/year vs. − 4.21 mL/min/1.73 m^2^/year (*p* = 0.698) in HCV infected and non-HCV infected mild CKD patients (**B**) and − 4.14 mL/min/1.73 m^2^/year vs. − 0.9 mL/min/1.73 m^2^/year (*p* < 0.001) in HCV infected and non-HCV infected moderate to severe CKD patients (**C**), respectively. The eGFR change was presented as mean and confidential interval (CI). Mild CKD denoted stage 1–2 CKD, and moderate to severe CKD, stage 3–5 CKD. CKD, chronic kidney disease; eGFR, estimated glomerular filtration rate; HCV, hepatitis C virus infection. * *p* < 0.05, annual eGFR of non-HCV versus HCV; # *p* < 0.05, eGFR slope of non-HCV versus HCV.

To further clarify the impact of HCV infection on the eGFR trajectories of patients according to the severity of renal dysfunction, we illustrated the differences of changes of slopes of eGFR stratified by HCV status and CKD stages during follow-up. The differences of eGFR slopes were greatest in normal renal function subjects but less prominent in mild CKD subjects stratified by HCV status (Fig. 4).

### HCV and 50% of eGFR decline from baseline value at third year

We performed an event-driven analysis using surrogate endpoint of 50% decline of eGFR from baseline value during 3 years of follow-up. From all of the normal renal function subjects, 26 of 167 (15.6%) HCV-infected patients but none of 779 (0%) non-HCV patients developed 50% decline of eGFR during follow-up. On the other hand, from overall CKD patients, 32 of 111 (28.9%) HCV-infected patients and 3 of 122 (2.5%) non-HCV patients developed 50% decline of eGFR at 3 years. The Firth logistic regression model revealed that HCV-infected normal renal function subjects had 80.54-fold increased risks of developing 50% decline of eGFR, after adjusting for age, gender, hypertension, diabetes, baseline renal function and UPCR, compared to non-infected counterparts. However, the risks of 50% eGFR decline associated with HCV were not significant in CKD patients after adjusting for many traditional risk factors (Table 3).

**Table 3:** Risks for the 50% decline in eGFR from the baseline value at 3-year of follow-up.

Variables	Normal renal function subjects				CKD patients			
	Crude		Adjusted*		Crude		Adjusted*	
	OR (95% CI)	*p*-value	OR (95% CI)	*p*-value	OR (95% CI)	*p*-value	OR (95% CI)	*p*-value
Age (per 1 year incremental)	1.06 (1.02–1.1)	0.006	1.08 (1.01–1.18)	0.016	0.99 (0.96–1.02)	0.456	0.96 (0.88–1.04)	0.317
Male Gender	2.28 (1.04–5.04)	0.04	4.43 (1.22–19.6)	0.023	1.21 (0.58–2.48)	0.603	1.17 (0.22–6.17)	0.846
Diabetes	33.03 (12.81–106.51)	< 0.001	11.29 (2.73–64.69)	< 0.001	4.47 (2.08–10.36)	< 0.001	2.98 (0.62–17.62)	0.172
Hypertension	1.03 (0.39–2.41)	0.95	3.26 (0.75–15.52)	0.114	0.93 (0.45–1.94)	0.835	0.86 (0.13–7.16)	0.879
Baseline eGFR (per 1 ml/min/1.73m^2^ incremental)	0.95 (0.92–0.97)	< 0.001	0.99 (0.95–1.03)	0.529	0.94 (0.91–0.96)	< 0.001	0.93 (0.88–0.97)	< 0.001
UPCR (mg/g)	1.0003 (1.0001–1.0005)	< 0.001	1.0001 (1–1.0004)	0.096	1.0007 (1.0004–1.0012)	< 0.001	1.0006(1.0003–1.0010)	< 0.001
HCV	278.9 (38.62–35,467.07)	< 0.001	80.54 (9.03–10,737.96)	< 0.001	13.96 (5.06–52.8)	< 0.001	1.54 (0.29–8.56)	0.603

*CKD* chronic kidney disease, *eGFR* estimated glomerular filtration rate, *HCV* hepatitis C virus, *OR* odd ratios, *UPCR* urine protein to creatinine ratio.

*Adjusted for age, gender, hypertension, diabetes mellitus, baseline eGFR, and UPCR.

## Discussion

Although the relationships between HCV infection and risk of CKD have been established in previous studies; however, the associated risks of renal progression have not been simultaneously explored in both normal renal function and CKD individuals. This 3-year prospective study has demonstrated that HCV infection has linked to increased risks of development of CKD and renal progression in the normal renal function subjects. However, the risk of renal progression associated with HCV in CKD patients may vary according to comorbidities and severity of renal dysfunction. The findings highlighted the importance of viral diagnosis with not only prognostic but also public health implications for preventing CKD and preserving renal function in the general population.

The incidence of CKD associated with HCV infection in our study was higher (217.3 per 1000 person-years) than previous estimates^5,6^. Differences in CKD definition, prevalence of HCV and variation of viral genotyping may explain the discrepancies observed among the diverse studies. The increases of risks of incident CKD and eGFR decline associated with HCV infection found in our study corroborated with the findings of some previous researches^3–7,9,14,20,22,29–31^ but inconsistent with others^24,25^. While some cross-sectional studies have applied cut-values of eGFR by using Modification of Diet in Renal Disease (MDRD) or CKD-EPI equation to define CKD^24,25^, others prospective studies have employed male-predominant participants^5^ or disease code-based registry claim data to ascertaining renal outcome rather than the changes of serum creatinine or eGFR slopes^4,6,29,30^. Consequently, serial measurements of serum creatinine and urine protein were largely lacking and may in part underestimate the exact incidence of CKD.

The faster eGFR decline observed in our HCV-infected normal renal function subjects than those infected CKD patients may further need investigation. The HCV can induce systemic inflammation and immune dysfunction triggering glomerular damage^19,32^. Nevertheless, the sharing of many risk factors may also play roles on the association between two disease entities^33–35^. However, the HCV infection remained significant risk factor of eGFR decline after adjusting for age, gender, hypertension, diabetes, baseline eGFR and UPCR in the normal renal function subjects of our study. In addition, interventional studies have indicated that reductions of viral load were associated with improvement of renal function in patients receiving antiviral treatment^7,29,36–39^, implicating an independent role of HCV in inducing renal damage from very early stage of disease.

Various subgroup analyses of CKD patients of this study have allowed better understanding of association of renal progression with HCV infection. The attenuation of risks of HCV on the renal progression of elderly, diabetic and hypertensive CKD patients was peculiar. The HCV interferes with insulin-signaling pathway, triggers epigenetics changes, modulates cellular gene expression, regulates circulating mi-RNA and promotes post-translational changes, leading to abnormal gluconeogenic and lipid storage process. Ultimately, it can result in insulin resistance, hyperglycemia and hepatic steatosis^40–42^. The association between HCV infection and diabetes mellitus has been extensively described^43,44^. Clinically, HCV-infected diabetes patients, who achieved sustained virologic response with direct-acting antiviral (DAA) therapy, have better glycemic control and less hypoglycemic drugs dosing than those who had treatment failure^45,46^. It is likely that the contribution of HCV infection to renal progression in CKD patients may in part masked by the coexistence of important comorbidities, considering all the metabolic, oxidative and inflammatory disarrangements associated with those diseases. Further experiments should be needed to elucidate the pathophysiological mechanisms of renal damage associated with HCV infection in the diverse primary kidney diseases and associated comorbidities.

Several limitations must also be addressed. First, the information regarding serum HCV RNA levels, viral genotypes and histological grading of renal or liver injury was lacking. HCV-related immune glomerulopathies could not be inferred from our study. However, we have excluded patients who received prior treatment of HCV from the study and may decrease the false-positive rate of active HCV infection and the antiviral drugs effects on renal function. Second, the findings were derived from a single ethnic population of high HCV prevalence, which may limit the generalizability of our findings to groups of different areas. Finally, many confounders, such as environmental exposures, nutrition and food styles, health-seeking behaviors and medications histories related to the development of CKD were not determined. Although such variables can be assumed as nondifferential misclassification in this study; however, serial measurements of renal function, simultaneous comparison of effects of HCV infection on the eGFR trajectories between normal renal function subjects and CKD patients, intermediate follow-up period of 36 months and multiple stratified/sensitivity analyses may strengthen the conjecture of our supposition.

In conclusion, individuals infected with HCV were at high risk of developing CKD. The HCV-infected normal renal function subjects were associated with greater risks of renal progression than the HCV-infected CKD patients. While effects of HCV infection on eGFR trajectory of diabetic or hypertensive CKD patients remains to be clarified; however, HCV infection may represent new modifiable CKD risk factor in the era of DAA therapy. The timely diagnosis of HCV infection and frequent kidney function monitoring in infected individuals should be recommended for routine clinical practice.

## Supplementary Information

Supplementary Information.

## Data Availability

All analytic data were incorporated into the article and the raw data underlying this article will be shared on reasonable request to the corresponding author.
