# Supplementary material for: Renal function trajectories in hepatitis C infection: differences between renal healthy and chronic kidney disease individuals
Source: Sci Rep. 2021 Aug 25;11:17197. doi: 10.1038/s41598-021-96782-x (PMC8387367; doi:10.1038/s41598-021-96782-x)
Supplement: Supplementary file 1 — Supplementary Information. [file 41598_2021_96782_MOESM1_ESM.docx]

**Table S1. Changes of eGFR in HCV and non-HCV infected normal renal function subjects**

| Population | HCV status | 1-year eGFR change | | *p* | | 2-year eGFR change | | | *p* | 3-year eGFR change | | | *p* | |  |  |
| --- | --- | --- | --- | --- | --- | --- | --- | --- | --- | --- | --- | --- | --- | --- | --- | --- |
| All patients |  |  | |  | |  | |  | | |  | |  | |  |  |
|  | **eGFR change** | |  | |  | |  | |  | | |  | |  | |  |
|  | HCV | -9.6 (-20.6 to -1) | | <0.001^#^ | | -15.4 (-32.3 to -5.1) | | <0.001^#^ | | | -18.3 (-32.4 to -6.1) | | <0.001^#^ | |  |  |
|  | Non-HCV | -1.7 (-9.9 to 7.9) | |  | | -0.9 (-9.6 to 7.3) | |  | | | -5.9 (-14.8 to 2.4) | |  | |  |  |
|  | **% of eGFR change from baseline** | | | | | | | | | | | | | |  |  |
|  | HCV | -11.6 (-22.8 to -1.4) % | | <0.001^#^ | | -14.2 (-28.5 to -2.4) % | | <0.001^#^ | | | -20 (-34.5 to -5.5) % | | <0.001^#^ | |  | |
|  | Non-HCV | -1.8 (-10.2 to 8.3) % | |  | | -1 (-10.1 to 7.7) % | |  | | | -6.2 (-14.8 to 2.8) % | |  | |  |  |
| Age, years |  |  | |  | |  | |  | | |  | |  | |  |  |
| 18-59 | **eGFR change** |  | |  | |  | |  | | |  | |  | |  |  |
|  | HCV | -8.1 (-21.2 to -0.5) | | <0.001^#^ | | -11.6 (-27.8 to -2.2) | | <0.001^#^ | | | -17.8 (-32.4 to -1) | | <0.001^#^ | |  |  |
|  | Non-HCV | -2.2 (-10.3 to 8) | |  | | -0.9 (-9.9 to 8.2) | |  | | | -6.4 (-16.1 to 2.4) | |  | |  |  |
|  | **% of eGFR change from baseline** | | | | | | | | | | | |  | |  |  |
|  | HCV | -8.9 (-23 to -0.4) % | | <0.001^#^ | | -14 (-28.9 to -2.3) % | | <0.001^#^ | | | -17.9 (-33.3 to -1.1) % | | <0.001^#^ | |  |  |
|  | Non-HCV | -2.2 (-10.4 to 8.3) % | |  | | -1 (-10 to 8.4) % | |  | | | -6.7 (-15.1 to 2.7) % | |  | |  |  |
| ≥60 | **eGFR change** |  | |  | |  | |  | | |  | |  | |  |  |
|  | HCV | -10.4 (-19.7 to -1.6) | | <0.001^#^ | | -12.2 (-24.2 to -2.3) | | <0.001^#^ | | | -17 (-31.8 to -6.4) | | <0.001^#^ | |  |  |
|  | Non-HCV | -0.5 (-7.6 to 7.9) | |  | | -1 (-9.6 to 5.8) | |  | | | -5.2 (-13.3 to 2.7) | |  | |  |  |
|  | **% of eGFR change from baseline** | | | | | | | | | | | |  | |  |  |
|  | HCV | -13 (-22.2 to -2.2) % | | <0.001^#^ | | -14.3 (-27.5 to -2.8) % | | <0.001^#^ | | | -20.5 (-35.6 to -8.5) % | | <0.001^#^ | |  |  |
|  | Non-HCV | -0.5 (-9.4 to 8.9) % | |  | | -1.2 (-10.6 to 6.9) % | |  | | | -5.6 (-13.8 to 3.2) % | |  | |  |  |
| Gender |  |  | |  | |  | |  | | |  | |  | |  |  |
| Men | **eGFR change** |  | |  | |  | |  | | |  | |  | |  |  |
|  | HCV | -12.8 (-20.5 to -1.4) | | <0.001^#^ | | -15.7 (-27.5 to -5.3) | | <0.001^#^ | | | -18.6 (-33.2 to -6.2) | | <0.001^#^ | |  |  |
|  | Non-HCV | -1.8 (-8.4 to 5.3) | |  | | -0.3 (-7.2 to 7.2) | |  | | | -2.9 (-10 to 2.8) | |  | |  |  |
|  | **% of eGFR change from baseline** | | | | | | | | | | | | | |  |  |
|  | HCV | -12.9 (-24.4 to -2.2) % | | <0.001^#^ | | -16.7 (-30.1 to -7.5) % | | <0.001^#^ | | | -24.2 (-36.8 to -9.7) % | | <0.001^#^ | |  |  |
|  | Non-HCV | -2 (-9.4 to 6) % | |  | | -0.4 (-7.8 to 7.8) % | |  | | | -3.6 (-10.8 to 3) % | |  | |  |  |
| Women | **eGFR change** |  | |  | |  | |  | | |  | |  | |  |  |
|  | HCV | -7.9 (-20.9 to -0.6) | | <0.001^#^ | | -10.9 (-24.7 to -0.7) | | <0.001^#^ | | | -16.4 (-29.9 to -4.8) | | <0.001^#^ | |  |  |
|  | Non-HCV | -1.4 (-10.6 to 10) | |  | | -1.3 (-11.5 to 7.4) | |  | | | -7.4 (-16.7 to 2.3) | |  | |  |  |
|  | **% of eGFR change from baseline** | | | | | | | | | | | |  | |  |  |
|  | HCV | -7.1 (-20.1 to -0.5) % | | <0.001^#^ | | -12 (-27.1 to -0.7) % | | <0.001^#^ | | | -17.9 (-32.7 to -4.8) % | | <0.001^#^ | |  |  |
|  | Non-HCV | -1.7 (-10.4 to 10.2) % | |  | | -1.2 (-11.4 to 7.6) % | |  | | | -7.7 (-15.8 to 2.4) % | |  | |  |  |
| Diabetes | **eGFR change** |  | |  | |  | |  | | |  | |  | |  |  |
|  | HCV | -13 (-22.4 to -2.5) | | <0.001^#^ | | -18.9 (-32.8 to -8.8) | | <0.001^#^ | | | -27.1 (-40.6 to -11.2) | | <0.001^#^ | |  |  |
|  | Non-HCV | -2.6 (-10.7 to 8.3) | |  | | 0.4 (-9.4 to 9.2) | |  | | | -4.3 (-15.6 to 4.5) | |  | |  |  |
|  | **% of eGFR change from baseline** | | | | | | | | | | | |  | |  |  |
|  | HCV | -13.4 (-28.9 to -3.7) % | | <0.001^#^ | | -21.7 (-40 to -11.8) % | | <0.001^#^ | | | -31.7 (-53.4 to -11.8) % | | <0.001^#^ | |  |  |
|  | Non-HCV | -3.6 (-11.4 to 8.3) % | |  | | 0.5 (-9.7 to 10) % | |  | | | -5.1 (-18.3 to 5.2) % | |  | |  |  |
| Non-Diabetes | **eGFR change** |  | |  | |  | |  | | |  | |  | |  |  |
|  | HCV | -5.3 (-18.3 to -0.3) | | <0.001^#^ | | -8.9 (-19.1 to -0.4) | | <0.001^#^ | | | -13.3 (-21.4 to -3.2) | | <0.001^#^ | |  |  |
|  | Non-HCV | -1.5 (-9.9 to 7.8) | |  | | -1.1 (-9.9 to 7.2) | |  | | | -5.9 (-14.8 to 2.4) | |  | |  |  |
|  | **% of eGFR change from baseline** | | | | | | | | | | | | | |  |  |
|  | HCV | -7.6 (-19.3 to -0.2) % | | <0.001^#^ | | -10.3 (-20.5 to -0.6) % | | <0.001^#^ | | | -15.8 (-25 to -4.5) % | | <0.001^#^ | |  |  |
|  | Non-HCV | -1.8 (-10.1 to 8.4) % | |  | | -1.1 (-10.2 to 7.5) % | |  | | | -6.2 (-14.5 to 2.8) % | |  | |  |  |
| HTN | **eGFR change** |  | |  | |  | |  | | |  | |  | |  |  |
|  | HCV | -13.6 (-20.6 to -4.8) | | <0.001^#^ | | -14.9 (-22.2 to -2.3) | | <0.001^#^ | | | -18.3 (-27.4 to -6.2) | | <0.001^#^ | |  |  |
|  | Non-HCV | -2.6 (-10.1 to 6.3) | |  | | -1.5 (-9.4 to 7.8) | |  | | | -4.2 (-14.8 to 3.9) | |  | |  |  |
|  | **% of eGFR change from baseline** | | | | | | |  | | |  | |  | |  |  |
|  | HCV | -16.3 (-22.1 to -5.2) % | | <0.001^#^ | | -15.6 (-27.3 to -2.4) % | | <0.001^#^ | | | -19.2 (-30.5 to -8.7) % | | <0.001^#^ | |  |  |
|  | Non-HCV | -2.5 (-10.7 to 7.1) % | |  | | -1.8 (-10.4 to 8.1) % | |  | | | -5 (-16.3 to 4.6) % | |  | |  |  |
| Non-HTN | **eGFR change** |  | |  | |  | |  | | |  | |  | |  |  |
|  | HCV | -8.5 (-20.9 to -0.6) | | <0.001^#^ | | -12 (-29.1 to -4.5) | | <0.001^#^ | | | -17.8 (-34.5 to -4.8) | | <0.001^#^ | |  |  |
|  | Non-HCV | -1.4 (-9.9 to 8.2) | |  | | -0.8 (-9.9 to 7.3) | |  | | | -6.2 (-14.9 to 2.3) | |  | |  |  |
|  | **% of eGFR change from baseline** | | | | | | |  | | |  | |  | |  |  |
|  | HCV | -11 (-24.2 to -0.5) % | | <0.001^#^ | | -14.3 (-30.3 to -4.4) % | | <0.001^#^ | | | -20.5 (-36.1 to -4.7) % | | <0.001^#^ | |  |  |
|  | Non-HCV | -1.7 (-10 to 8.5) % | |  | | -0.9 (-9.8 to 7.6) % | |  | | | -6.6 (-14.5 to 2.4) % | |  | |  |  |

eGFR changes were expressed as median (IQR) in mL/min/1.73 m^2^. ^#^ *p*-value using Mann-Whitney U test.

Abbreviations: CKD, chronic kidney disease; eGFR, estimated glomerular filtration rate; HCV, hepatitis C virus.

**Table S2. Changes of eGFR in HCV and non-HCV infected CKD patients**

| Population | HCV status | 1-year eGFR change | *p* | 2-year eGFR change | *p* | 3-year eGFR change | *p* |
| --- | --- | --- | --- | --- | --- | --- | --- |
| All patients |  |  |  |  |  |  |  |
|  | **eGFR change** |  |  |  |  |  |  |
|  | HCV | -6 (-11.2 to -1.5) | <0.001^#^ | -9.3 (-14.5 to -3.5) | <0.001^#^ | -11.8 (-20.4 to -6.5) | 0.003^#^ |
|  | Non-HCV | -1.9 (-9.2 to 7.1) |  | -3.2 (-13.1 to 4.5) |  | -6.6 (-17.9 to -1) |  |
|  | **% of eGFR change from baseline** | | | | | | |
|  | HCV | -15.9 (-26.1 to -6.7) % | <0.001^#^ | -24.9 (-41.9 to -9.2) % | <0.001^#^ | -33.9 (-50.7 to -17.9) % | <0.001^#^ |
|  | Non-HCV | -2.1 (-11.6 to 10) % |  | -3.3 (-18 to 6.6) % |  | -9.6 (-21.3 to -0.9) % |  |
| Age, years |  |  |  |  |  |  |  |
| 18-59 | **eGFR change** |  |  |  |  |  |  |
|  | HCV | -7 (-14.6 to -1.6) | 0.004^#^ | -14.6 (-22.5 to -6.5) | <0.001^#^ | -15.1 (-26.4 to -6.7) | 0.003^#^ |
|  | Non-HCV | 3.3 (-9.5 to 10.8) |  | -0.2 (-9.1 to 6.7) |  | -2.7 (-15.3 to 3.3) |  |
|  | **% of eGFR change from baseline** | | | | | | |
|  | HCV | -22.6 (-37.8 to -7.8) % | <0.001^#^ | -42.5 (-62.4 to -19.3) % | <0.001^#^ | -41.5 (-53.8 to -24.1) % | 0.001^#^ |
|  | Non-HCV | 3.1 (-11.6 to 12.6) % |  | -0.2 (-12.5 to 7.5) % |  | -3.7 (-14.4 to 4.2) % |  |
| ≥60 | **eGFR change** |  |  |  |  |  |  |
|  | HCV | -6 (-10.7 to -1.4) | 0.004^#^ | -8.7 (-12.7 to -3.4) | 0.086^#^ | -10.6 (-18.7 to -6.4) | 0.147^#^ |
|  | Non-HCV | -2.1 (-9.1 to 3) |  | -5.1 (-14.3 to 3.1) |  | -7.6 (-18.4 to -2.1) |  |
|  | **% of eGFR change from baseline** | | | | | |  |
|  | HCV | -15.8 (-25 to -6) % | <0.001^#^ | -21.5 (-35.2 to -8.6) % | <0.001^#^ | -30.1 (-49.2 to -17.5) % | <0.001^#^ |
|  | Non-HCV | -2.6 (-14.5 to 5.3) % |  | -8.2 (-18.8 to 4.9) % |  | -12.9 (-25.8 to -2.9) % |  |
| Gender |  |  |  |  |  |  |  |
| Men | **eGFR change** |  |  |  |  |  |  |
|  | HCV | -6.3 (-11.7 to -2.3) | 0.015^#^ | -9.3 (-15.7 to -2.7) | 0.013^#^ | -10.4 (-19.6 to -7) | 0.001^#^ |
|  | Non-HCV | -2.8 (-9.3 to 2.3) |  | -1.7 (-14.1 to 3.1) |  | -4.6 (-13.2 to 1.1) |  |
|  | **% of eGFR change from baseline** | | | | | |  |
|  | HCV | -16.4 (-30.9 to -7.3) % | <0.001^#^ | -20.5 (-38.8 to -7.7) % | 0.001^#^ | -28.6 (-50.2 to -17.3) % | <0.001^#^ |
|  | Non-HCV | -4.4 (-15.4 to 5.7) % |  | -2.5 (-20 to 5.4) % |  | -6.5 (-19.5 to 1.7) % |  |
| Women | **eGFR change** |  |  |  |  |  |  |
|  | HCV | -5.3 (-10.9 to -0.8) | 0.003^#^ | -10.3 (-14.4 to -3.8) | 0.012^#^ | -12.3 (-20.7 to -6.4) | 0.152^#^ |
|  | Non-HCV | -0.6 (-9.2 to 8.6) |  | -3.5 (-12.8 to 5.8) |  | -9.4 (-18.5 to -1.2) |  |
|  | **% of eGFR change from baseline** | | | | | |  |
|  | HCV | -15.1 (-25.2 to -5.3) % | <0.001^#^ | -27.8 (-43.3 to -10) % | <0.001^#^ | -39.4 (-51.3 to -21.7) % | <0.001^#^ |
|  | Non-HCV | -1.4 (-11.2 to 10.7) % |  | -4 (-16.2 to 6.9) % |  | -12.7 (-21.8 to -1.1) % |  |
| Diabetes | **eGFR change** |  |  |  |  |  |  |
|  | HCV | -6.5 (-11.7 to -2.5) | 0.057^#^ | -10.7 (-16.3 to -3.5) | 0.986^#^ | -13.4 (-22 to -8.7) | 0.621^#^ |
|  | Non-HCV | -2.7 (-11.2 to 2.4) |  | -9.9 (-20.2 to -0.7) |  | -13.8 (-24.5 to -4.3) |  |
|  | **% of eGFR change from baseline** | | | | | | |
|  | HCV | -19 (-27.6 to -7.5) % | <0.001^#^ | -27.7 (-43.8 to -9.6) % | <0.013^#^ | -39.4 (-52.6 to -24.6) % | <0.001^#^ |
|  | Non-HCV | -5.3 (-18.5 to 4.7) % |  | -14.5 (-31.8 to -0.6) % |  | -18.9 (-32.9 to -3.9) % |  |
| Non-Diabetes | **eGFR change** |  |  |  |  |  |  |
|  | HCV | -5.4 (-9.4 to -0.4) | 0.005^#^ | -8.8 (-13 to -3.3) | <0.001^#^ | -9.9 (-17.2 to -4.8) | 0.011^#^ |
|  | Non-HCV | 0 (-8.2 to 9) |  | -0.9 (-8.8 to 6) |  | -5 (-17.2 to 1.2) |  |
|  | **% of eGFR change from baseline** | | | | | | |
|  | HCV | -14.8 (-24.4 to -2.1) % | <0.001^#^ | -21.2 (-35.6 to -8.9) % | <0.001^#^ | -27.6 (-45.5 to -11.7) % | <0.001^#^ |
|  | Non-HCV | 0 (-10.6 to 10.7) % |  | -1.1 (-11.6 to 7) % |  | -6.6 (-15.6 to 2) % |  |
| HTN | **eGFR change** |  |  |  |  |  |  |
|  | HCV | -5.8 (-9.9 to -1.3) | 0.002^#^ | -8.9 (-14.2 to -3.6) | 0.045^#^ | -11.7 (-21 to -6.4) | 0.169^#^ |
|  | Non-HCV | -1.5 (-8 to 6.6) |  | -5.4 (-14.9 to 1.2) |  | -7.7 (-18.5 to -2.2) |  |
|  | **% of eGFR change from baseline** | | | |  |  |  |
|  | HCV | -14.8 (-24.5 to -5.3) % | <0.001^#^ | -24.8 (-41.1 to -10) | <0.001^#^ | -31.4 (-49.2 to -18) | <0.001^#^ |
|  | Non-HCV | -2.1 (-13.5 to 8) % |  | -6.5 (-22.2 to 1.9) % |  | -13.1 (-26.9 to -3.3) % |  |
| Non-HTN | **eGFR change** |  |  |  |  |  |  |
|  | HCV | -8.3 (-13.4 to -1.8) | 0.012^#^ | -9.7 (-14.3 to -0.5) | 0.011^#^ | -12 (-18.9 to -6.2) | 0.013^#^ |
|  | Non-HCV | -0.6 (-10.4 to 9.7) |  | 2 (-12.4 to 7.8) |  | -4.6 (-17.4 to 5.2) |  |
|  | **% of eGFR change from baseline** | | | |  |  |  |
|  | HCV | -20.9 (-32.8 to -6.5) % | <0.001^#^ | -21.6 (-43.9 to -0.7) % | <0.001^#^ | -35.7 (-58.1 to -11.1) | <0.001^#^ |
|  | Non-HCV | -1.4 (-11.6 to 12.7) % |  | 1.7 (-13.2 to 11.8) % |  | -4.2 (-15.6 to 7) % |  |

eGFR changes were expressed as median (IQR) in mL/min/1.73 m^2^. ^#^ *p*-value using Mann-Whitney U test.

Abbreviations: CKD, chronic kidney disease; eGFR, estimated glomerular filtration rate; HCV, hepatitis C virus.
